# Gingivitis efficacy of a 0.454% w/w stannous fluoride dentifrice: a 24-week randomized controlled trial

**DOI:** 10.1186/s12903-020-01079-6

**Published:** 2020-03-26

**Authors:** C. R. Parkinson, K. R. Milleman, J. L. Milleman

**Affiliations:** 1 GlaxoSmithKline Consumer Healthcare, Weybridge, KT13 0DE UK; 2 Salus Research, Inc., Fort Wayne, IN USA

**Keywords:** Stannous fluoride, Dentifrice, Gingivitis, Plaque

## Abstract

**Background:**

Plaque-induced gingivitis can be prevented and treated with regular effective oral hygiene, principally via mechanical cleaning with regular toothbrushing. To complement the mechanical plaque removal, antimicrobial ingredients can be incorporated into dentifrices to inhibit the growth of plaque. This study aimed to evaluate and compare gingivitis and the proportion of subjects moving between gingivitis severity (< 10, > 10 < 30, > 30% bleeding sites), and plaque reduction, following twice daily use of an experimental non-aqueous 0.454% weight/weight (w/w) stannous fluoride (SnF_2_) dentifrice, compared to a negative control dentifrice over 12 and 24 weeks.

**Method:**

This was a single-center, examiner-blinded, randomized, stratified, two-treatment arm, parallel group, 24-week clinical study in healthy adult volunteers with moderate gingivitis. At baseline, after abstaining from toothbrushing overnight, subjects underwent MGI (modified gingival index), BI (bleeding index) and PI (plaque index) assessments. Eligible subjects, who met the inclusion/exclusion criteria, were stratified based on gender and baseline mean MGI score (Low ≤2.00 /High > 2.00) and randomized to treatment. Following randomization, subjects underwent a thorough dental prophylaxis and flossing. After 12 and 24 weeks of twice daily brushing with their allocated treatment, subjects returned to the site (with overnight plaque, having abstained from oral hygiene procedures for 8 h prior to visit) for MGI, BI and PI assessments. Treatment effect was evaluated by comparing the MGI, BI and PI scores.

**Results:**

One hundred and twenty-nine subjects were screened; 98 subjects were randomized and 90 subjects completed the study. Statistically significant differences between treatments, in favour of the 0.454% stannous fluoride dentifrice were observed, compared to the negative control dentifrice, for all outcome measures (MGI, BI, bleeding sites and PI at weeks 12 and 24 *p* < 0.0001). At 24 weeks, 71% of subjects in the 0.453% SnF_2_ treatment group demonstrated < 10% of bleeding sites.

**Conclusion:**

A dentifrice containing 0.454% w/w SnF_2_ was shown to be superior to a standard dentifrice in controlling gingivitis and supra-gingival plaque, over a 24-week period. Over two thirds of subjects in the 0.454% SnF_2_ treatment group demonstrated a level of bleeding sites potentially representative of “clinical periodontal health” (< 10%) following a dental prophylaxis and 24 weeks of product use.

**Trial registration:**

This study was retrospectively registered at ClinicalTrials.gov, on 11th Oct. 2019 (NCT04123665).

## Background

Plaque induced gingivitis is reported to have a high prevalence worldwide [1], and it is widely agreed to be a pre-requisite of periodontitis [2].

Plaque induced gingivitis occurs if dental plaque is left to accumulate, initially at or below the gingival margin, leading to a loss of symbiosis between the dental biofilm and the host’s immune-inflammatory response. Gingivitis is initially characterized by subtle changes of the gingiva without loss of periodontal attachment, however as the disease progresses the symptoms may become more obvious to the patient; they may be aware of symptoms that include bleeding on tooth brushing, gingival swelling and redness, and they may also report tenderness and halitosis in the case of established forms [2].

Importantly, plaque-induced gingivitis can be prevented and treated with regular effective oral hygiene, principally via mechanical cleaning with regular toothbrushing [3]. To complement such mechanical plaque removal, antimicrobial ingredients can be incorporated into dentifrices to inhibit the growth of plaque, particularly in areas of the mouth less accessible to the toothbrush [4].

Stannous fluoride (SnF_2_) is a broad-spectrum antimicrobial agent that has demonstrated efficacy in daily use dentifrices for the control of dental plaque and treatment of gingivitis [5]. More specifically, the stannous (II) ion (Sn [II]) has been shown to be the bioactive species that exerts the antiplaque effect by reducing bacterial biomass/virulence and inhibiting bacterial metabolism [5–7]. Clinically, the largest improvements in gingival health have been observed with dentifrice formulations that have employed methods to stabilise and maintain stannous in the bioactive Sn [II] state, until the point of use [5, 8]. One such method to maintain stannous in the bioactive state is to formulate the compound in an anhydrous base [9].

This study is one of a series comparing the efficacy and tolerability of twice-daily brushing with a stannous fluoride-containing dentifrice, stabilized in a non-aqueous base. In agreement with guidance for evaluating the gingivitis efficacy of chemotherapeutics, this study forms the second of two long-term comparative studies conducted by independent investigators (defined as clinical institutions and investigators unique to each other) and utilising disjoint study populations [10].

The aim of this study was to evaluate the gingivitis efficacy of a dentifrice containing 0.454% w/w SnF_2_, stabilized in a non-aqueous base, after 24 weeks twice daily brushing, and to confirm the findings of a previously reported study [11]. An additional objective was to explore the treatment response by evaluating the proportion of subjects moving between gingivitis severity category, as expressed by number of bleeding sites at the subject level (< 10, > 10 < 30, > 30% bleeding sites).

## Methods

This was an examiner-blind, randomized, stratified, two-treatment, parallel group, 24 week, clinical study in healthy adult volunteers with moderate gingivitis. The study was carried out at Salus Research Inc., Fort Wayne, Indiana, US, with the study protocol approved by an independent review board (‘U.S. Investigational Review Board’, Miami, FL 33143) IRB number: U.S. IRB2013SRI/04. It was performed in accordance with the requirements specified in the Declaration of Helsinki and relevant local laws and regulations. All eligible subjects provided written informed consent before initiation of study procedures. There were no protocol amendments.

### Subjects

Subjects were ≥ 18 years old, in good general physical health with ≥20 natural teeth. At screening/baseline subjects were required to have moderate gingivitis (in the opinion of the clinical examiner and with a whole mouth MGI score between 1.75 and 2.30), a mean whole mouth PI score ≥ 1.5, and a minimum of 40 tooth surfaces where at least 50% was gradable for each clinical index (third molars, orthodontically banded/bonded, fully crowned, extensively restored, or grossly carious teeth were not included in the tooth count). Exclusion criteria included: pregnancy; breast feeding; allergy/intolerance to the study materials; current smokers or who had quit within the 6 months prior to the study; use of smokeless forms of tobacco; taking, or had taken, in the 14 days prior to the baseline visit, antibiotics, anti-inflammatory medication or a systemic medication that could affect gingival condition; participation in another clinical trial or use of an investigational oral care product within 30 days of baseline visit. Dentition exclusions included: current active caries or periodontitis that could compromise the study or oral health of the subject; restorations in a poor state of repair; partial dentures or orthodontic appliances; teeth bleaching within 12 weeks of screening; use of a chlorhexidine mouthwash within 14 days of baseline.

### Objectives

The primary objective of this study was to evaluate the gingivitis efficacy of a dentifrice containing 0.454% w/w SnF_2_, stabilized in a non-aqueous base, after a dental prophylaxis and 24 weeks twice daily brushing, as assessed by bleeding index (BI). Secondary objectives were to explore between treatment differences by BI at 12 weeks, and modified gingival index (MGI), number of bleeding sites, and plaque index (PI) at 12 and 24 weeks. An exploratory objective was to explore the treatment response by evaluating the proportion of subjects moving between gingivitis severity category, as expressed by the number of bleeding sites at the subject level (< 10, > 10 < 30, > 30% bleeding sites).

### Procedures and assessments

At the screening visit, subjects gave their written informed consent to participate in the study. Demographic, medical history and concomitant medications were recorded, followed by an oral examination and a gingival assessment that included a gross oral soft tissue (OST) examination, an oral hard tissue visual examination and assessment of dentition exclusions and gingival status. Within 28 days of the screening visit, eligible subjects returned to the site for the baseline visit with overnight plaque (subjects abstained from oral hygiene from 21:00 the night before the visit).

Subjects underwent a full OST examination and assessments of gingival inflammation (MGI), gingival bleeding (BI) and supra-gingival plaque (PI), carried out by the same examiner throughout the study to control for inter-examiner variability.

Eligible subjects (those with a mean MGI score between 1.75–2.30, a mean whole mouth PI score ≥ 1.5 and who met all inclusion and exclusion criteria) were stratified based on gender and baseline mean whole mouth MGI scores (moderate gingivitis between 1.75 and 2.30, stratified by Low ≤2.00/High > 2.00 MGI score) to ensure a balance in gingivitis across both treatment groups and randomized to one of two treatments according to a schedule provided by the Biostatistics Department of the study sponsor. All subjects received a dental prophylaxis using a conventional non-fluoride prophylaxis paste followed by flossing and removal of residual plaque by dental polishing to bring their teeth to zero plaque. This was checked by a second examiner to ensure complete removal of plaque.

Subjects were assigned one of two study dentifrices: a 0.454% SnF_2_ dentifrice containing 0.454% w/w SnF_2_ (Sensodyne Complete Protection, GSK Consumer Healthcare, Weybridge, UK; US marketed dentifrice) or a negative control dentifrice containing 1000 ppm fluoride as sodium monofluorophosphate (SMFP) (Colgate Cavity Protection, Colgate-Palmolive Co., New York, US; US marketed dentifrice). Colgate Cavity Protection was chosen as the negative control as it was considered representative of a regular toothpaste (not indicated for gingivitis). Subjects were instructed to apply a full ribbon of dentifrice to the head of a supplied manual toothbrush and brush their teeth in their usual manner for one timed minute twice daily (morning and evening). Study products were overwrapped to mask their identity as far as possible. The study examiner, study statistician, data management staff and other employees of GSK Consumer Healthcare or site staff who could have influenced study outcomes were blinded to product allocation.

#### Modified gingival index and bleeding index

Gingivitis was assessed using the MGI, BI and number of bleeding sites (as bleeding index score of 1 or 2). The MGI is a noninvasive evaluation of visual changes of severity and extent of gingivitis. MGI was assessed on the facial and lingual surfaces of two sites of each scorable tooth (papillae and margin/7–7 in each arch). Two scores were recorded bucally/labially (papilla and margin) and two scores lingually/palatally (papilla and margin). The MGI scoring system ranges from 0 (absence of inflammation) to 4 (severe inflammation), as described by Lobene *et al* [12]. The BI assesses the number of bleeding points elicited on probing as a measure of gingival condition. Gingivae were air dried and then a ball-ended ‘community periodontal index of treatment needs probe’ was inserted into the gingival crevice to a depth of approximately 1 mm and ran around the tooth, gently stretching the epithelium. The BI was assessed on the facial and lingual gingival surfaces of each scorable tooth (7–7 in each arch). Three scores were recorded bucally/labially (distal, body, mesial sites) and three scores lingually/palatally. The BI scoring system was as follows: 0 = no bleeding after 30 s; 1 = bleeding upon probing after 30 s; 2 = immediate bleeding observed. The number of bleeding sites was calculated for each subject as the number of sites with a BI of 1 or 2 across all evaluable tooth sites [13].

#### Plaque index

The six-site modification of the Turesky Modification of the Quigley Hein Index (PI) was employed to assess plaque on all natural, gradable teeth [14]. Plaque was first disclosed using a dye solution (Gum Red-Cote®, Sunstar Americas, Inc., Schaumburg, IL, US). For assessment, each tooth was divided into six areas including the mesiofacial, facial, distofacial, mesiolingual, lingual, and distolingual surfaces. Disclosed plaque was scored on a scale of 0 (no plaque) to 5 (plaque covering 2/3 or more of the crown of the tooth) [14].

#### Adverse events

All spontaneously-reported adverse events (AEs) or abnormalities in the OST examination were recorded from the screening visit until 5 days after the last study product administration. The relationship between the occurrence of each AE and the product was assessed by the investigator using clinical judgment and graded as mild, moderate or severe. Treatment emergent AEs were reported for the safety population (all randomized subjects who received the study treatment).

### Statistical methods

#### Sample size

It was planned to screen enough subjects such that 100 would be randomized to treatment to ensure a total of 88 subjects (44 per treatment group) completed the week 24 assessment. With 44 subjects per treatment group the study was calculated to have 90% power to detect a difference between treatments of 0.07 units in BI after 24 weeks of treatment, assuming a standard deviation of 0.10, with a 0.05 two-sided significance level.

The primary population for the assessment of efficacy was the Intent-to-Treat (ITT) population, defined as those who received their study treatment and had at least one post-baseline efficacy measurement. The primary outcome variable was whole mouth mean BI at 24 weeks, calculated by taking the mean of the BI scores over all evaluable sites. Secondary efficacy variables were whole mouth mean BI at 12 weeks and, whole mouth mean MGI, bleeding sites, PI at all sites and at interproximal sites only, and BI and MGI by high/low MGI subgroup, at 12 and 24 weeks.

The BI, MGI, PI, and number of bleeding sites at 12 and 24 weeks were compared between treatments using analysis of covariance (ANCOVA). For all but PI, the model included factors for treatment group and gender, with baseline BI and MGI as covariates. The MGI stratification factor was not included in the model as the actual value was included as a covariate. In the analysis of number of bleeding sites, the baseline whole mouth mean BI score was included as a covariate. Model assumptions of normality and homogeneity of variance were tested and found not to be violated.

Subgroup analyses of the BI and MGI variables were performed based on the low and high MGI levels at stratification using ANCOVA with factors for treatment group, gender, MGI stratification and treatment by MGI stratification interaction, with baseline BI and MGI as covariates. For PI scores (overall and interproximal sites), the model included factors for treatment group, gender and MGI stratification, with the baseline PI score (overall or interproximal as appropriate) as a covariate.

For each subject, the percentage of bleeding sites at each time point was calculated as the number of sites with a bleeding score of 1 or 2, divided by the total number of bleeding sites assessed (multiplied by 100). The percentage of bleeding sites was categorized into four groups: < 10%, 10–20%, 20–30 and > 30%.

All tests were two sided and performed at the 5% significance level under a null hypothesis of no difference between treatments. Model assumptions were checked and deemed to be acceptable.

Subjects who withdraw from the study early were included in the statistical analysis up to the point of when they withdraw. The drop-out rate over the 24-week study period was expected to be low (< 10%) and therefore there was no provision for imputation for missing data.

## Results

### Demographic and baseline characteristics

**Fig. 1:**
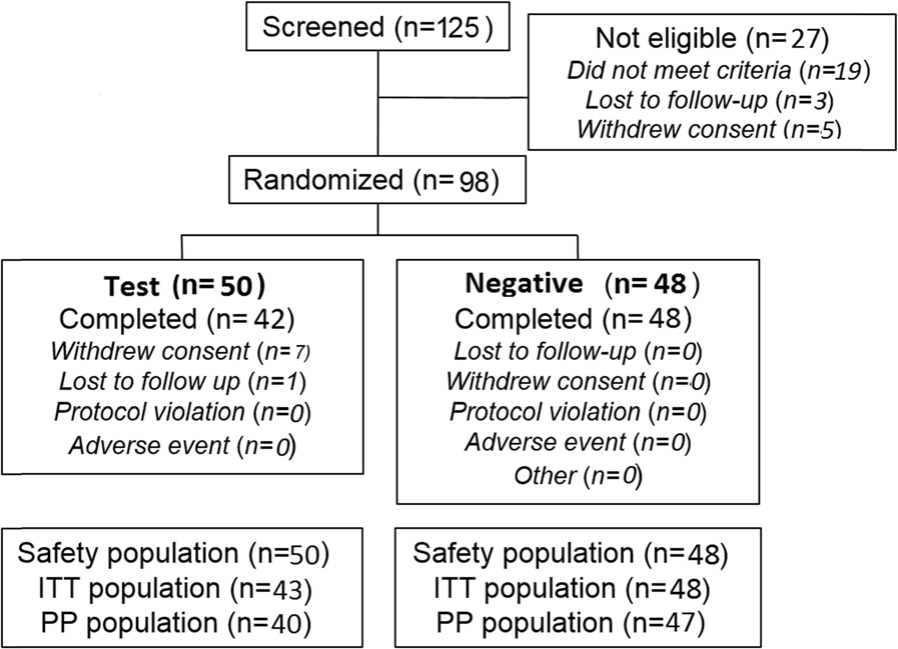
Study flow. (ITT, intent to treat; PP, per protocol)

The first subject was enrolled on 28th May 2013 with the last subject completing the study on 11th December 2013. Of the 125 subjects screened, 98 were randomized to treatment and 90 completed the study (Fig. 1). Demographics (safety population) are presented in Table 1. Age ranged from 18 to 70 years, with a mean age of 39.2 years; the majority were female (66.3%). At baseline, 10.2% were male with a baseline mean whole mouth MGI in the low strata (≤2.00), 23.5% were male with a baseline mean whole mouth MGI in the high strata (> 2.00); 34.7% were female with a baseline mean whole mouth MGI in the low strata (≤2.00) and 31.6% were female with a baseline mean whole mouth MGI in the high strata (> 2.00). The two treatment groups were observed to be well balanced for demographic and baseline characteristics.

**Table 1:** Baseline demographics and characteristics (safety population)

	0.454% SnF_**2**_ (*n* = 50)	Negative(*n* = 48)
**Gender, n (%)**		
Female	34 (68.0)	31 (64.6)
Male	16 (32.0)	17 (35.4)
**Race, n (%)**		
White	43 (86.0)	39 (81.3)
Black/African-American	4 (8.0)	8 (16.7)
American Indian/Alaska native	1 (2.0)	0 (0.0)
Asian	0 (0.0)	1 (2.1)
Multiple	2 (4.0)	0 (0.0)
**Mean age, years (SD)**	39.5 (14.55)	38.8 (12.44)
**Strata (baseline MGI; Sex) n (%)**		
≤ 2.0; Male	5 (10.0)	5 (10.4)
> 2.0; Male	11 (22.0)	12 (25.0)
≤ 2.0; Female	18 (36.0)	16 (33.3)
> 2.0; Female	16 (32.0)	15 (31.3)

A total of 91 subjects were included in the efficacy analysis, based on the ITT population. A separate PP population was not performed because the difference in subject numbers between the ITT and PP populations was less than the predefined threshold of 10% different.

#### Efficacy results

After a dental prophylaxis and 24 weeks treatment a statistically significantly reduction in whole mouth gingival bleeding (BI) was observed for the 0.454% SnF_2_ dentifrice compared to the Negative Control dentifrice (primary outcome) (Table 2). Statistically significant differences, in favour of the 0.454% SnF_2_ dentifrice, were also observed for secondary outcome measures including mean BI at week 12, mean MGI, and plaque index at weeks 12 and 24 (Table 2).

**Table 2:** Efficacy endpoints: difference in adjusted means at each visit (ITT population)

	Mean (SE)		Difference^**a**^ (95% CI)	% diff^**b**^.	***p***-value
	SnF_**2**_	Negative			
**Bleeding Index**					
Baseline	0.30 (0.021)	0.31 (0.027)	–	–	–
Week 12	0.11 (0.017)	0.23 (0.016)	−0.13 (− 0.17, − 0.08)	−54.6	< 0.0001
Week 24	0.11 (0.013)	0.19 (0.012)	− 0.07 (− 0.11, − 0.04)	−39.5	< 0.0001
**Bleeding Sites**					
Baseline	28.93 (1.916)	29.15 (2.223)	–	–	–
Week 12	12.26 (1.596)	23.92 (1.596)	−11.72 (−16.08, −7.35)	−48.9	< 0.0001
Week 24	12.69 (1.562)	20.19 (1.331)	−7.08 (−10.63, −3.53)	−35.4	0.0002
**Modified Gingival Index**					
Baseline	2.04 (0.024)	2.03 (0.020)	–	–	–
Week 12	1.42 (0.045)	1.87 (0.042)	−0.45 (− 057, − 0.33)	−24.0	< 0.0001
Week 24	1.45 (0.048)	1.78 (0.045)	−0.33 (− 0.46, − 0.20)	−18.5	< 0.0001
**Plaque Index**					
Baseline	3.17 (0.064)	3.06 (0.065)	–	–	–
Week 12	2.56 (0.053)	2.93 (0.050)	−0.37 (− 0.51, − 0.22)	−12.5	< 0.0001
Week 24	2.53 (0.057)	2.86 (0.054)	−0.33 (− 0.48, − 0.17)	−11.4	< 0.0001
**Interproximal Plaque Index**					
Baseline	3.29 (0.057)	3.18 (0.060)	–	–	–
Week 12	2.78 (0.045)	3.08 (0.042)	−0.29 (− 0.42, − 0.17)	−9.6	< 0.0001
Week 24	2.75 (0.050)	3.02 (0.047)	−0.28 (− 0.42, − 0.14)	−9.2	< 0.0001

^a^From ANCOVA analysis; difference is 0.454% SnF_2_ minus the negative control; negative difference favors 0.454% SnF_2_

^b^Percentage calculated as (difference/adjusted mean of reference)*100

**Fig. 2:**
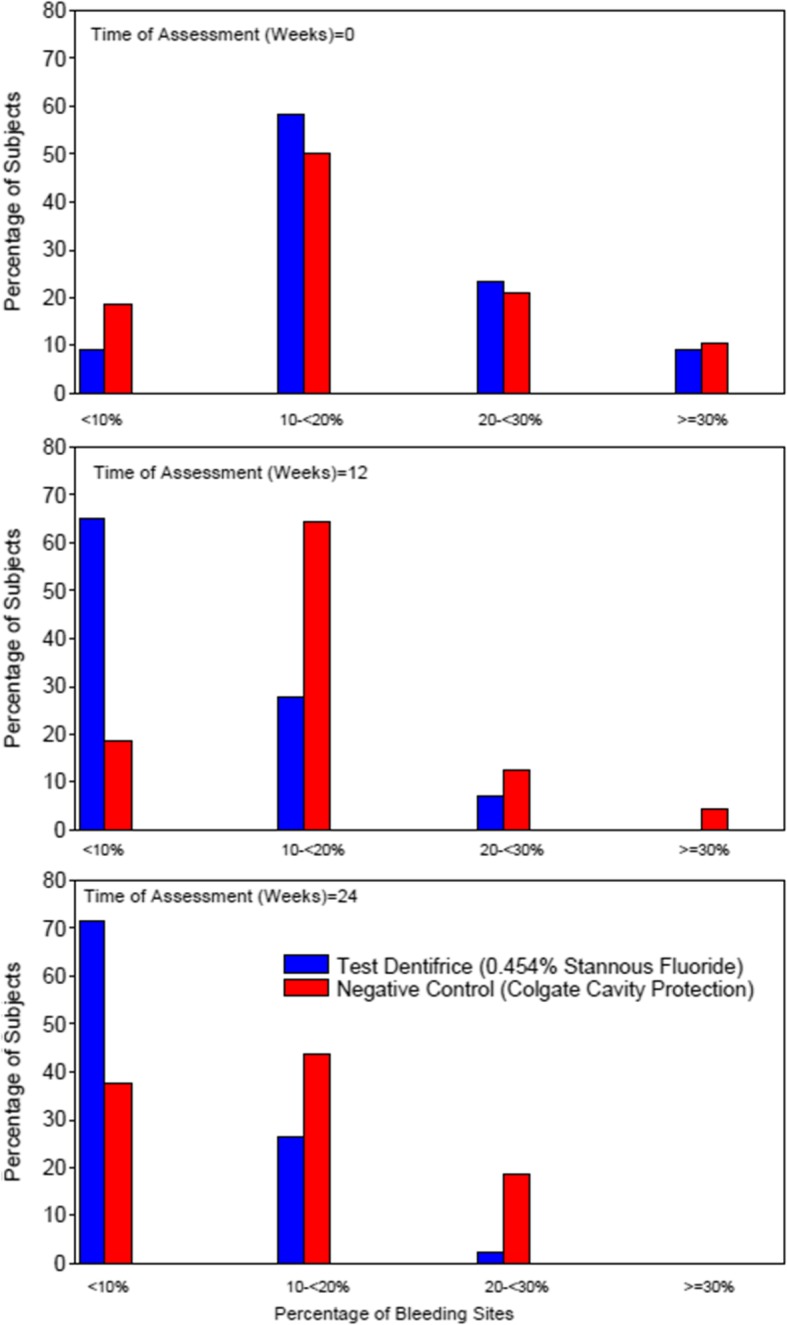
% distribution of subjects with < 10, > 10 < 30, and > 30% bleeding sites by treatment and timepoint. The percentage of bleeding sites at each timepoint was calculated as the number of sites with a bleeding score of 1 or 2, expressed as a percentage of the total number of bleeding sites assessed

Consistent with BI, there were statistically significantly fewer bleeding sites in the treatment group using the 0.454% SnF_2_ dentifrice compared to the negative control dentifrice at weeks 12 and 24 (Table 2). At weeks 12 and 24, approximately twice as many subjects in the 0.454% SnF_2_ dentifrice group demonstrated < 10% of bleeding sites (71% at week 24), compared to the negative control group (38%, at week 24). The distribution of subjects with < 10, > 10 < 30, and > 30% bleeding sites by treatment group at each timepoint is given in Fig. 2.

Results for the low and high MGI sub-groups generally resembled the overall analysis with differences between treatments in favour of the 0.454% SnF_2_ dentifrice for BI and MGI at both 12 and 24 weeks. In terms of magnitude, larger differences in favour of the 0.454% SnF_2_ dentifrice were observed for BI in the ‘high MGI baseline>2.0’ subgroup (treatment difference in BI of 0.09 at 24 weeks), compared to BI in the ‘low MGI baseline ≤2.0’ subgroup (treatment difference in BI of 0.05 at 24 weeks); and for MGI in the ‘high MGI baseline>2.0’ subgroup (treatment difference in MGI of 0.35 at 24 weeks), compared to MGI in the ‘low MGI baseline ≤2.0’ subgroup (treatment difference in MGI of 0.3 at 24 weeks).

#### Safety results

One subject reported one treatment emergent AE of mouth ulceration in the negative Control dentifrice group. The AE was mild in intensity and not consider to be treatment related.

## Discussion

In this study, the dentifrice containing 0.454% w/w SnF_2_ was statistically significantly superior to a negative control dentifrice in controlling signs of gingivitis (gingival bleeding and visual signs of gingival inflammation) and plaque, following 12 and 24 weeks of use. The magnitude of difference observed for the measures of gingival health of 39.5% (BI), 35.5% (bleeding sites), 18.5% (MGI) at 24 weeks between the 0.454% SnF_2_ and negative control treatments (Table 2) are considered clinically significant and confirm the results of a series of previously reported clinical studies of 3 to 6 months in duration, conducted on an identical formulation [11, 15].

Evaluation of the distribution of subjects by extent of gingivitis (number of bleeding sites) by treatment group, over the treatment period, demonstrates progressive clinically relevant improvements in gingival health for subjects in the 0.454% SnF_2_ treatment group, over the treatment period. In 2018, a revision to the 1999 classification system for gingival diseases was published [16–19]. The 2018 revision proposed 4 categories of periodontal health, described by Lang et al. [18] as *“(1) pristine periodontal health defined as a total absence of clinical inflammation and physiological immune surveillance on a periodontium with normal support (no attachment or bone loss)”;* “*(2) clinical periodontal health characterized by an absence or minimal levels of clinical inflammation in a periodontium with normal support*”; “*(3) periodontal disease stability, in a reduced periodontium*”, and “*(4) periodontal disease remission/control, in a reduced periodontium*” [18]. Within category 2 (potentially representative of the population in this study), the extent of gingivitis, in terms of the number of gingival sites exhibiting inflammation was further characterized and described by Murakami et al. [19], as “*incipient*”, “*localized*” or “*generalized*”; whereby “*incipient*” gingivitis is described as “*only a few sites affected by mild inflammation, expressed as mild redness and/or a delayed and broken line of bleeding rather than edema or an immediate unbroken line of bleeding on probing*”; “*Localized*” gingivitis is described as “*when < 30% of the teeth are affected by gingival inflammation*”; and “*generalized*” “*when ≥30% of the teeth are affected by gingival inflammation*” [19]. An important aspect of the classification system is that “*incipient*” gingivitis has been described in Maurkami [19] and others “*as a condition that is part of a spectrum of clinical health*” [19]. Trombelli at al [17] further defined “*only a few sites*” for “clinical periodontal health”. In the context of epidemiological reseach, “few sites” is considered to be less than 10% sites of inflammation (on an otherwise periodontally healthy patient) [17].

In this study, approximately 70% of subjects in the 0.454% SnF_2_ dentifrice group demonstrated < 10% of bleeding sites, after a dental prophylaxis and 24 weeks use. Acknowledging the limitations of a clinical trial setting, and that recruitment and assessment in this study was not specifically conducted according to the 2018 classification system, nevertheless the significant shift of subjects observed in the SnF_2_ treatment group may be representative of a shift from “localised/generalised gingivitis” to “incipient gingivitis” or approaching “the continuum of clinical health”, as reported in the 2018 revision of the classification system for gingival diseases [17–19].

While such patients periodontal health could potentially be describe as “a condition that is part of a spectrum of clinical health”, it is important to consider that these patients are at greater risk of rapidly regressing back to localised gingivitis, and therefore it is important that they continue to practice effective plaque control, with dentifrices designed to complement the mechanical plaque removal of toothbrushing, and regular and appropriate standard of professional dental care.

## Conclusion

The results of this 24 week clinical study confirm the findings of previously reported clinical studies on non-aqueous stabilised SnF_2_ dentifrice [11, 15]. Clinically relevant reduction in the number of bleeding sites observed for the 0.454% SnF_2_ treatment group (in conjunction with a dental prophylaxis) may represent a meaningful improvement in gingivitis, with over two thirds of subjects achieving 10% or less bleeding sites, that is potentially representative of the spectrum of clinical (periodontal) health. Both treatments were generally well tolerated.

## Data Availability

The datasets analyzed during the current study are available from the corresponding author on request.

## Abbreviations

AE: Adverse events
ANCOVA: Analysis of covariance
BI: Saxton & Van der Ouderra bleeding index
BL: Baseline
CI: Confidence interval
Diff: Difference
GSKCH: GSK Consumer Healthcare
ITT: Intent-to-treat
MGI: Lobene Modified Gingival Index
n (%): Number (percent)
n: Number
nAE: Number of adverse events
NaF: Sodium fluoride
OST: Oral soft tissue
PI: Lobene Plaque index
PP: Per protocol
SMFP: Sodium monofluorophosphate
SnF_2_: Stannous fluoride
SD: Standard deviation
